# Conjugates of a Photoactivated Rhodamine with Biopolymers for Cell Staining

**DOI:** 10.1155/2014/285405

**Published:** 2014-10-14

**Authors:** Sergei Yu. Zaitsev, Mikhail N. Shaposhnikov, Daria O. Solovyeva, Valeria V. Solovyeva, Albert A. Rizvanov

**Affiliations:** ^1^Moscow State Academy of Veterinary Medicine and Biotechnology, Akad. Skryabin Street 23, Moscow 109472, Russia; ^2^Kazan Federal University, Kremlevskaya Street 18, Kazan 420008, Russia

## Abstract

Conjugates of the photoactivated rhodamine dyes with biopolymers (proteins, polysaccharides, and nucleic acids) are important tools for microscopic investigation of biological tissue. In this study, a precursor of the photoactivated fluorescent dye (PFD) has been successfully used for staining of numerous mammalian cells lines and for conjugate formation with chitosan (“Chitosan-PFD”) and histone H1 (“Histone H1.3-PFD”). The intensive fluorescence has been observed after photoactivation of these conjugates inside cells (A431, HaCaT, HEK239, HBL-100, and MDCK). Developed procedures and obtained data are important for further application of novel precursors of fluorescent dyes (“caged” dyes) for microscopic probing of biological objects. Thus, the synthesized “Chitosan-PFD” and “Histone H1-PFD” have been successfully applied in this study for intracellular transport visualization by fluorescent microscopy.

## 1. Introduction

Design of various conjugates of fluorescent dyes and their nonfluorescent precursors with proteins, polysaccharides, nucleic acids, and other biomolecules are the rapidly growing fields at the “edge” of molecular biology and chemistry, biophysics, and biochemistry [1–3]. Such substances can serve both for fundamental study [4–9] and for applications in materials sciences and biotechnology [10–13]. A novel approach is the molecular design and study of the “caged” fluorescent dyes that are capable of penetrating of cell membrane and thereby suitable for ultrahigh resolution optical microscopy [4–8, 13–17]. The “caged” fluorescent dyes are especially useful for visualization of biologically active substances (BAS), intracellular macromolecules, and various biological processes (the self-organization, molecular recognition, transport, etc.) as well as promising materials for microscopy [10–13]. The main efforts in studying the so-called “caged” fluorescent dyes are concentrated in the field of rhodamines modifications. For example, the introduction of the compact photosensitive groups (thioamides or N-nitrosamines) in the rhodamine molecule leads to the preparation of the photoactivated fluorescent dyes (PFDs) in the “caged” (closed) form which becomes more hydrophobic and promotes the membrane permeability. PFD can be transferred to fluorescent (open) form by photoactivation with wavelength ≥ 375 nm [14–17]. Varying the power of the illuminating light and its location, one can easily vary the number and spatial arrangement of the newly formed fluorescent “probes,” then track their movement, and determine the shape and positioning of cellular and subcellular objects labeled by such dyes. The recent studies [17, 18] demonstrate also the possibility of using fluorescent dyes for obtaining 2D and 3D structures of the cells and intracellular objects. Due to their high photostability and excellent photophysical properties, rhodamine dyes can be especially useful as laser dyes, fluorescent standards, and trackers of macromolecular movement, in addition to the abovementioned application as cell fluorescent probes [4–8, 10–13]. Amazing precursors of rhodamine dyes with advanced propertieshave recently been synthesized and introduced that are particularly useful for microscopic investigation of biological objects by fluorescent microscopy [19–23].

The main goal of this work was to study multifunctional precursor's conjugates of the fluorescent dye (PFD) with chitosan and histone H1 in order to visualize the transport of these biopolymers into the cells of different types.

## 2. Materials and Methods

### 2.1. Materials

**Figure 1:**
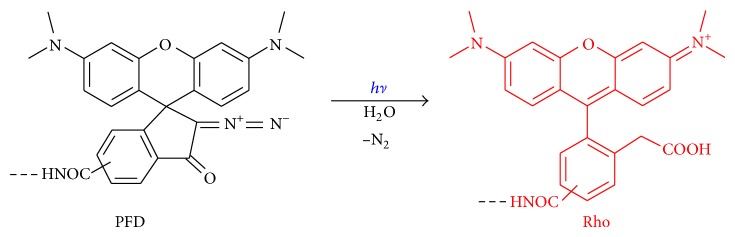
Chemical structures of precursors of fluorescent dyes PFD and the rhodamine dyes Rho formed by photoactivation.

The synthesis and properties of the “caged” PFD dye (Figure 1) have been described earlier [8, 9, 17, 20]. The “Hoechst 33342” dye has been used for permanent florescence labeling (without photoactivation) of the cell nucleus as reference. Cells were cultivated and handled in the Institute of Bioorganic Chemistry, Russian Academy of Sciences: human breast carcinoma HBL-100, human keratinocytes HaCaT, human epithelial carcinomas A431 and canine kidney epithelial cells MDCK. Primary cell cultures of human lymphocytes were isolated by standard procedure [18–21]. Cell trackers: MitoTracker Green FM and Hoechst 33342, were purchased from Invitrogen and Sigma-Aldrich, respectively.

### 2.2. Measurements

The following PFD concentrations were used: PFD solutions from 0.02 to 10 *μ*g/mL in the cell culture media from the stock solutions in DMF at 10 mg/mL and in DMSO at 2 mg/mL. Cells were incubated for 30 min at 37°C in CО_2_ incubator. During this incubation (15 min after beginning), the nuclei staining dye Hoechst 33342 was added to cell cultures. After incubation, cells were fixed for 15 min and washed with phosphate buffer (pH 7.0). These samples were mounted on glass slides using Mowiol 4.88 (Calbiochem, Darmstadt, FRG) and stored at 4°C overnight for the complete polymerization. All samples were analyzed using confocal microscope Eclipse TE2000 (Nikon) with EZ-C1 3.90 Free Viewer software. The images were compared by color intensity in different channel using software “ImageJ” and plugin “Colocalization Finder.”

A chitosan with a molecular weight of 50 kDa (degree of deacetylation of 96%) and PFD-NHS were used for the “Chitosan-PFD” conjugate preparation. In particular, chitosan amino groups were acylated with PFD-NHS to form an amide bond and N-hydroxysuccinimide. PFD-813 and PFD-NHS with chitosan solutions (for comparison and verification) and the “Chitosan-PFD” conjugate were added to the plate for 1 hour pregrown on coverslips cell monolayers at final concentration of 2.2 *μ*g/mL. Cells on coverslips were washed with phosphate buffer and mounted using Mowiol 4.88 on slides after incubation for 1 hour.

**Figure 2:**
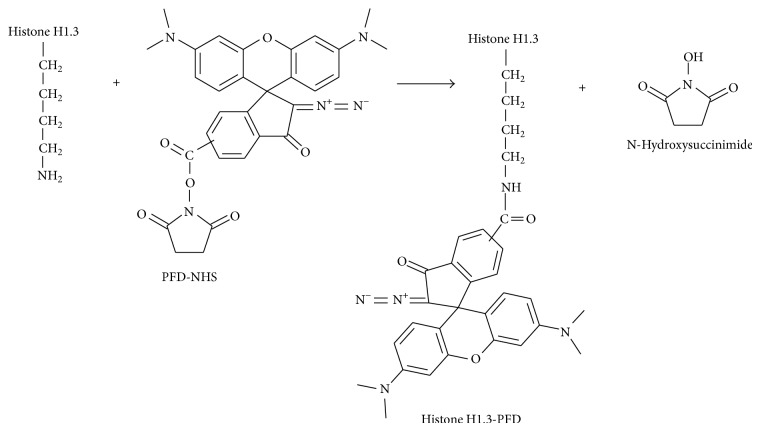
Scheme of the conjugate of histone H1.3 with PFD (“Histone H1.3-PFD”) synthesis.

The conjugate of histone H1.3 (provided by Human Stem Cell Institute, Moscow, Russia) with PFD (tetramethylrhodamine derivative), capable of bright fluorescence in the red region of the spectrum (after photoactivation), was synthesized by standard procedure: (1) 0.5 mg PFD-NHS was dissolved in 50 *μ*L DMSO and added to 200 mL of bicarbonate buffer (50 mM), containing 2 mg of histone H1.3; (2) the resulting obtained mixture was stirred for 1.5 hours at 20°C; (3) the mixture was neutralized by 0.1 N acetic acid and distilled water was added to 500 mL final volume. Succinimide group of PFD promotes formation of the covalent bonds with primary amino groups (lysine and arginine residues) of histone H1.3 to form the conjugate “Histone H1-PFD” (Figure 2). The conjugate “Histone H1-PFD” was purified from the mixture using chromatography on Sephadex G-25 filled column.

## 3. Results and Discussion

**Figure 3:**
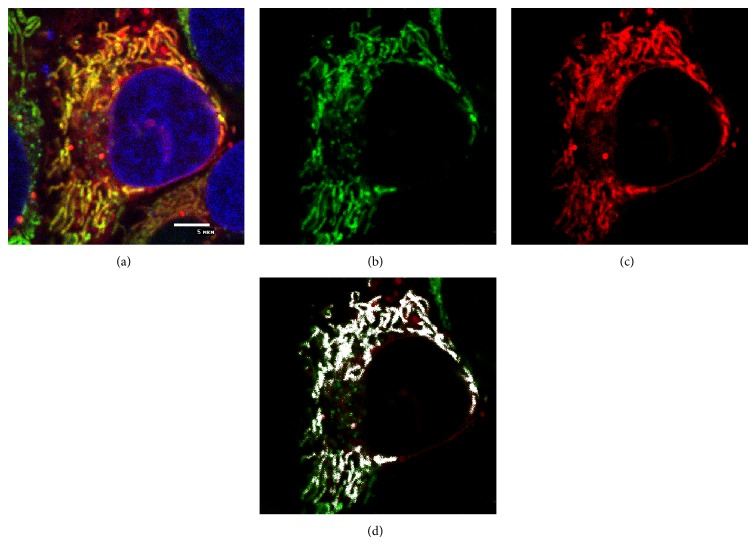
Microphotographs of the A431 cells stained by PFD (novel dye called Rho813 after photoactivation) and the standard dyes MitoTracker Green FM and Hoechst 33342. (a) Image obtained with 3 channels (for each dye) simultaneously. (b) The cell image obtained only with green channel (MitoTracker Green FM). (c) The cell image obtained only with red channel (Rho813). (d) The cell image obtained with overlapping green and red channels that gave white spots (“colocalization” areas of red and green fluorescence).

Some data of similar PFDs (structure, physical-chemical properties, and interactions with lipids in model membranes) was published in the recent papers [9, 18, 20]. In the previous study, the method of cell staining by the most promising precursor PFD of fluorescent dye Rho813 was developed and described in detail [19, 23]. This dye was tested with various samples of the human cell cultures (human breast carcinoma HBL-100, human keratinocytes HaCaT, human epithelial carcinomas A431, and primary cell cultures of human lymphocytes) as well as with canine kidney epithelial cells MDCK. Investigation of such cell staining by PFD (precursor) and Rho813 (dye) using fluorescence microscopy allows one to find the optimal sample concentrations in various solvents (technical notes concerning this procedure have been published earlier [19–23]). Optimal concentration of PFD solutions for cell staining was 5 *μ*g/mL in all cases. High resolution images of various cell types were obtained after light-induced activation of PFD (Figure 3).

The best micrographs were obtained after photoactivation of “live” cells stained by PFD. The advantages of such methodology (making preparations of “live” cells) are the following: the absence of the fixing agent (formaldehyde solution); replacement of the washed buffer by fresh culture medium; and the exclusion of a polymerizable material (e.g., “Mowiol”). The only disadvantage of such methodology is the necessity of fast measurements of such samples because of the rapid evaporation of the culture medium between the glass plates.

As can be concluded from the previous experiments, PFD is concentrated mainly in the intracellular organelles of the “live” cells (Figure 3) as compared to the fixed cells [19]. In our opinion, the main subcellular structures stained by PFD may be mitochondria and lysosomes. For example, A431 cells were stained by PDF813 and simultaneously with MitoTracker Green FM and Hoechst 33342 (Figure 3). After PFD photoactivation in the cell (by 405 nm wavelength light), three channels of confocal microscopy were used: for Hoechst 33342, blue colored nucleus; for MitoTracker Green FM, green colored mitochondria; and for Rho813, red colored organelles (PFD) (Figure 3). The separate green and red channels for the same cell image are shown in Figures 3(b) and 3(c). After their comparison (Figures 3(b) and 3(c)), the overlapping (merged) image with white spots (“colocalization” areas of red and green fluorescence) was obtained (Figure 3(d)). White spots occupied about 72.6% of the whole area fluoresced in red in Figure 3(c). The small green and red spots occupied the majority of the photoactivated area to prove the mitochondria staining. The same procedure was used for the detection of lysosomes in the same cells (data not shown). Thus, the novel precursor of fluorescent dye PFD has been successfully tested with various cell cultures.

**Figure 4:**
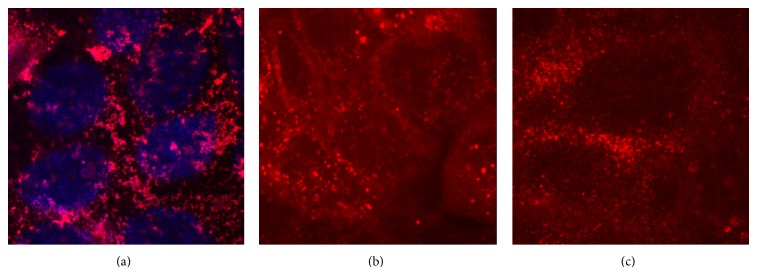
HaCaT cells stained by conjugate “Chitosan-PFD” (conc. 2.2 mg/mL) (a), the mixture of PFD (conc. 5 *μ*g/mL) and chitosan (2.2 *μ*g/mL) (b), and PFD-NHS (conc. 5 *μ*g/mL) and chitosan (2.2 *μ*g/mL) (c) after photoactivation (image dimensions, 36 by 36 *μ*m).

The rhodamine derivatives can be used to study biopolymer transport using fluorescent microscopy. Chitosan is important biopolymer for modeling and applied studies of the BAS transport in various cells. Chitosan heterogeneous clusters in the cells were observed around the nucleus after photoactivation (Figure 4(a)). The images of cells stained with the PFD (or PFD-813-NHS) in the presence of chitosan are shown in Figures 4(b) and 4(c) for comparison. In the case of last two mixtures, dye distribution inside cell was more uniform (Figures 4(b) and 4(c)) as compared to the conjugate (Figure 4(a)). This can be a consequence of the free dye distribution (separately from the chitosan) in the case of the abovementioned mixtures. Moreover, the synthesized conjugate of chitosan with PFD molecules (“Chitosan-PFD”) is suitable for chitosan imaging inside the cells using laser scanning confocal microscopy. The discussed conjugate is just one example of such PFD application in the study of molecular transport visualized by fluorescent microscopy.

**Figure 5:**
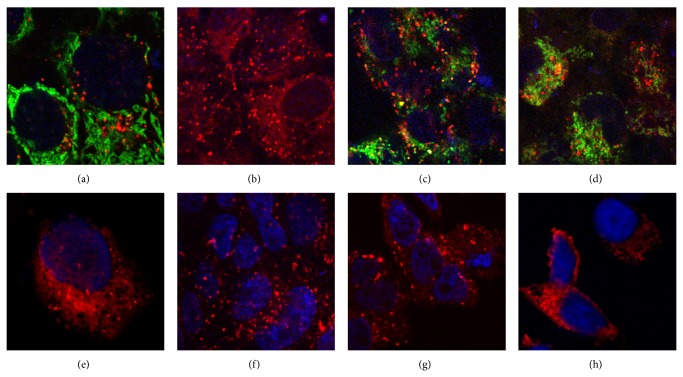
Images of the following cell lines: A431 ((a), (e)), MDCK ((b), (f)), HEK239 ((c), (g)), HBL-100 ((d), (h)) stained by the conjugate of histone H1.3 with PFD (“Histone H1-PFD”) for the “live” (images (a)–(d)) and fixed (images (e)–(h)) cells (image dimensions, 42 by 42 *μ*m).

Another promising example is the conjugate “Histone H1-PFD” (Figure 2). It is important that the amounts up to 0.25 mg per mL of histone H1.3 and conjugate “Histone H1.3-PFD” are not toxic to HeLa cell culture (data not shown). The data on transportation and intracellular localization of the conjugate was obtained for the following cell lines: HEK293, A431, HeLa, HBL-100, and MDCK, which were incubated for 1 hour with 30 *μ*g/mL “Histone H1-PFD”. Microphotographs of “native” and fixed cells in order to evaluate the intracellular distribution of the conjugate were obtained using the confocal microscope (Figure 5).

It is important to emphasize that the obtained conjugate was able to penetrate into all studied cells and interact with major organelles. 50 *μ*g of MitoTracker Green FM has been dissolved in 75 *μ*L DMSO up to concentration of 1 mM and 1 *μ*L of the obtained solution has been diluted in 99 *μ*L water (only 11 *μ*L of the final solution has been applied in each vial). It is found in all cases that the obtained conjugates after cell penetration can be seen as small aggregates (size about 1 *μ*m or less) which are clearly distinguishable in all studied cells (Figures 5(a)–5(d)). More valuable data was obtained by comparative experiments with conjugate “Histone H1-PFD” staining the “live” (Figures 5(a)–5(d)) and fixed (Figures 5(e)–5(h)) cells. In order to study localization of the conjugate “Histone H1-PFD” in the cells, the blue nuclear dye (Hoechst 33342) and green mitochondrial dye (MitoTracker Green FM) have been photographed with a 1-second interval between channels (Figure 5). It can be assumed from the clearly seen features in the microphotographs (Figure 5) that after one hour of incubation the conjugate is mainly localized in secondary lysosomes (according to the size and shape of the structures, etc.) but only in the small amount is localized in mitochondria. It is unusual that in the MDCK cell line the conjugate is nonspecifically distributed in the “live” cells (Figure 5(b)). In contrast, only nonspecific distribution can be observed for conjugate staining of the fixed cell of different cell lines (A431, HEK239, and HBL-100) (Figures 5(d), 5(g), and 5(h)). Thus, a conjugate is more evenly distributed in the fixed cells, as compared to “live” cells.

The authors anticipated that the resulting conjugate can be used for further study of biopolymer (proteins, polysaccharides, nucleic acids, etc.) and drug delivery systems into human and animal cells.

## 4. Conclusions 

Properties of the novel PFD before and after photoactivation to fluorescent rhodamine derivative (Rho813) have been characterized in the fixed and “live” cells. The PFD demonstrated better cell staining properties as compared to other rhodamine caged dyes. Such dyes are very stable during photoactivation, have intensive fluorescence even at low dye concentration, and easily penetrate various cell types, selectively staining some organelles (mainly mitochondria and lysosomes). Conjugates of chitosan or histone H1.3 with PFD were obtained as examples to investigate the distribution of different biopolymers within cells. The intensive fluorescence has been observed after the photoactivation of these conjugates inside different cell cultures (A431, HaCaT, HEK239, HBL-100, and MDCK). The synthesized “Chitosan-PFD” and “Histone H1-PFD” have been successfully applied in this study for intracellular transport visualization by fluorescent microscopy. The PFDs potential for nanoscale optical resolution renders them particularly suited for special tasks in biology and bionanotechnology and human and animal medicine.
